# Identifying highly heritable brain amyloid phenotypes through mining Alzheimer’s imaging and sequencing biobank data

**Published:** 2022

**Authors:** Jingxuan Bao, Zixuan Wen, Mansu Kim, Xiwen Zhao, Brian N. Lee, Sang-Hyuk Jung, Christos Davatzikos, Andrew J. Saykin, Paul M. Thompson, Dokyoon Kim, Yize Zhao, Li Shen, Alzheimer’s Disease Neuroimaging Initiative

**Affiliations:** 1 Department of Biostatistics, Epidemiology and Informatics University of Pennsylvania Perelman School of Medicine, Philadelphia, PA 19104, USA; 2 Department of Biostatistics, Yale University School of Public Health, New Haven, CT 06511, USA; 3 Center for Biomedical Image Computing and Analytics, Department of Radiology University of Pennsylvania Perelman School of Medicine, Philadelphia, PA 19104, USA; 4 Department of Radiology and Imaging Sciences, Indiana University School of Medicine, Indianapolis, IN 46202, USA; 5 Imaging Genetics Center, Stevens Institute for Neuroimaging and Informatics University of Southern California School of Medicine, Marina del Rey, CA 90292, USA

**Keywords:** Heritability, Quantitative trait, Brain imaging genetics, Alzheimer’s Disease

## Abstract

Brain imaging genetics, an emerging and rapidly growing research field, studies the relationship between genetic variations and brain imaging quantitative traits (QTs) to gain new insights into the phenotypic characteristics and genetic mechanisms of the brain. Heritability is an important measurement to quantify the proportion of the observed variance in an imaging QT that is explained by genetic factors, and can often be used to prioritize brain QTs for subsequent imaging genetic association studies. Most existing studies define regional imaging QTs using predefined brain parcellation schemes such as the automated anatomical labeling (AAL) atlas. However, the power to dissect genetic underpinnings under QTs defined in such an unsupervised fashion could be negatively affected by heterogeneity within the regions in the partition. To bridge this gap, we propose a novel method to define highly heritable brain regions. Based on voxelwise heritability estimates, we extract brain regions containing spatially connected voxels with high heritability. We perform an empirical study on the amyloid imaging and whole genome sequencing data from a landmark Alzheimer’s disease biobank; and demonstrate the regions defined by our method have much higher estimated heritabilities than the regions defined by the AAL atlas. Our proposed method refines the imaging endophenotype constructions in light of their genetic dissection, and yields more powerful imaging QTs for subsequent detection of genetic risk factors along with better interpretability.

## 1. Introduction

Brain imaging genetics,^1–3^ an emerging and rapidly growing research field, studies the relationship between genetic variations and brain imaging quantitative traits (QTs) to gain new insights into the phenotypic characteristics and genetic mechanisms of the brain. With recent advances in multimodal neuroimaging and high throughput genotyping and sequencing technologies, researchers are able to investigate the mechanisms behind biological and/or pathological pathways from genetic determinants to brain structure and function and then to the human cognition, behaviors and disorders. In particular, the availability of rapidly growing brain imaging and genomics biobanks has led to a large body of literature concerning methodological developments and biomedical applications in brain imaging genetics (e.g.,^4–10^).

Heritability^11^ is an important measurement to quantify the proportion of the observed variance in an imaging QT that is explained by genetic factors, and can often be used to prioritize brain QTs for subsequent imaging genetic association studies. Currently, many existing heritability studies couple the atlas-based brain parcellations with imaging measures to define the brain QTs. However, most of the brain parcellations are predefined based on the anatomical knowledge and/or structural and functional annotation without embracing genetic explanation for the corresponding regions of interest (ROIs). Thus, in brain imaging genetics, regional imaging QTs are often defined based on these predefined brain parcellation schemes such as the automated anatomical labeling (AAL) atlas.^12^ However, the power to dissect genetic underpinnings under QTs defined in such an unsupervised fashion could be negatively affected by heterogeneity within the regions in the partition.

To bridge this gap, we propose a novel method to define highly heritable brain regions. We employ the Genome-wide Complex Trait Analysis (GCTA),^13^ which is a widely used statistical tool for heritability estimation. It utilizes the individual-level genetic data to construct the genetic relationships among subjects and uses a mixed linear model to quantify the environmental effect and genetic effect for the variation of phenotypic quantitative traits. In this work, we propose a data driven method to group highly heritable voxel-level imaging QTs according to their significance level estimated from GCTA and their spatial location.

From voxelwise heritability estimates, we extract brain regions containing spatially connected voxels with high heritability based on a user-specified threshold. To evaluate our proposed method, we apply our data driven method to the amyloid imaging data and the whole genome sequencing (WGS) data from the Alzheimer’s Disease Neuroimaging Initiative (ADNI) cohort,^14–16^ which is a landmark Alzheimer’s disease biobank. We demonstrate the regions defined by our method have much higher estimated heritabilities than the regions defined by the AAL atlas. Our proposed method refines the imaging endophenotype constructions in light of their genetic dissection, and yields more powerful imaging QTs for subsequent detection of genetic risk factors along with better interpretability.

The rest of this paper is organized as follows. We introduce our heritability estimation method in Section 2, discuss our data and materials in Section 3, describe our experimental workflow in Section 4, present and discuss our results in Section 5, and conclude the paper in Section 6.

## 2. Method

Given an imaging QT, its heritability^11^ is defined to be the proportion of its total phenotypic variance that is explained by the aggregated genetic effect captured by pedigree information or all the single nucleotide polymorphisms (SNPs) on a genotyping or sequencing array.^1^ Since ADNI is a population study instead of a family study, subjects are unrelated and no pedigree information is available for heritability analysis. However, there is SNP-based genotyping and WGS data in ADNI. Therefore we focus on estimating heritability using the SNP data.

In particular, we use the following linear mixed effects (LME) model to estimate SNP-based heritability:^13^

(1) y=Xβ+Wu+ε,

where *y* is an *N* × 1 vector of quantitative traits (QTs) with *N* being the number of subjects, *β* is the vector of fixed effects, *X* is the matrix of confounding variables (i.e., age, sex and population structure represented by first 10 principal components in our experiments), *u* is a vector of SNPs effects with $u\sim N\left(0,\;I\sigma_{u}^{2}\right)$, where *I* is an identity matrix, and *W* is a standardized genotype matrix. $\varepsilon \sim N\left(0,\;I\sigma_{\varepsilon }^{2}\right)$ is the error term. The genetic relationship matrix (GRM) between individuals is defined as $A=\frac{WW^{\prime }}{M}$, where *M* is the number of SNPs.

In fact, heritability^17^ is formally defined as the proportion of phenotypic variation that is due to variation in genetic values. In the LME model, it can be computed as

(2) $$h^{2}=\frac{M\sigma_{u}^{2}}{M\sigma_{u}^{2}+\sigma_{\varepsilon }^{2}}=\frac{M\sigma_{u}^{2}}{\sigma_{y}^{2}}.$$

The LME model has already been implemented in the GCTA tool.^13^ Thus, in this work, we directly use GCTA to compute heritability for all the studied QTs.

## 3. Materials

Data used in the preparation of this article were obtained from the ADNI database.^14–16^ Specifically, the genetic data used in our analysis were the ADNI whole genome sequencing (WGS) data downloaded from the Alzheimer’s Disease Sequencing Project (ADSP) website at https://www.niagads.org/adsp.^18,19^ All the imaging and other data were downloaded from the ADNI website at https://adni.loni.usc.edu.

The ADNI was launched in 2003 as a public-private partnership, led by Principal Investigator Michael W. Weiner, MD. The primary goal of ADNI has been to test whether serial magnetic resonance imaging (MRI), positron emission tomography (PET), other biological markers, and clinical and neuropsychological assessment can be combined to measure the progression of mild cognitive impairment (MCI) and early Alzheimer’s disease (AD). The up-to-date information about the ADNI is available at https://www.adni-info.org.

Our WGS data contains 31,200,009 SNPs with 1,546 samples. We performed the quality control (QC) using the following criteria: minor allele frequency (MAF) > 0.001; call rate (GENO) > 98%; identity-by-descent (IDB) estimates < 0.25; Hardy-Weinberg test at 10^−6^ significance threshold; missing rate per person (MIND) < 0.05; excluding the outliers from Heterozygosity X missingness plot. After QC, 15,363,329 SNPs and 1,546 samples are preserved.

Out of 1,546 subjects with the WGS data available after QC, 1,047 participants have complete [^18^F]florbetapir (AV45) PET data (measuring amyloid burden) and are included in our analysis. Table 1 shows the participant characteristics; and our analysis includes 333 cognitively normal (CN), 384 mild cognitive impairment (MCI), and 330 AD subjects. For these AV45 PET scans, the data was registered to the Montreal Neurological Institute space, and the standard uptake value ratio was computed by intensity normalization using the cerebellar curs reference region. ROI-level AV45 measures were extracted based on the AAL atlas,^12^ where 116 ROI-level QTs were obtained by averaging all the voxel-level measures within each ROI.

## 4. Experimental Workflow

**Fig. 1:**
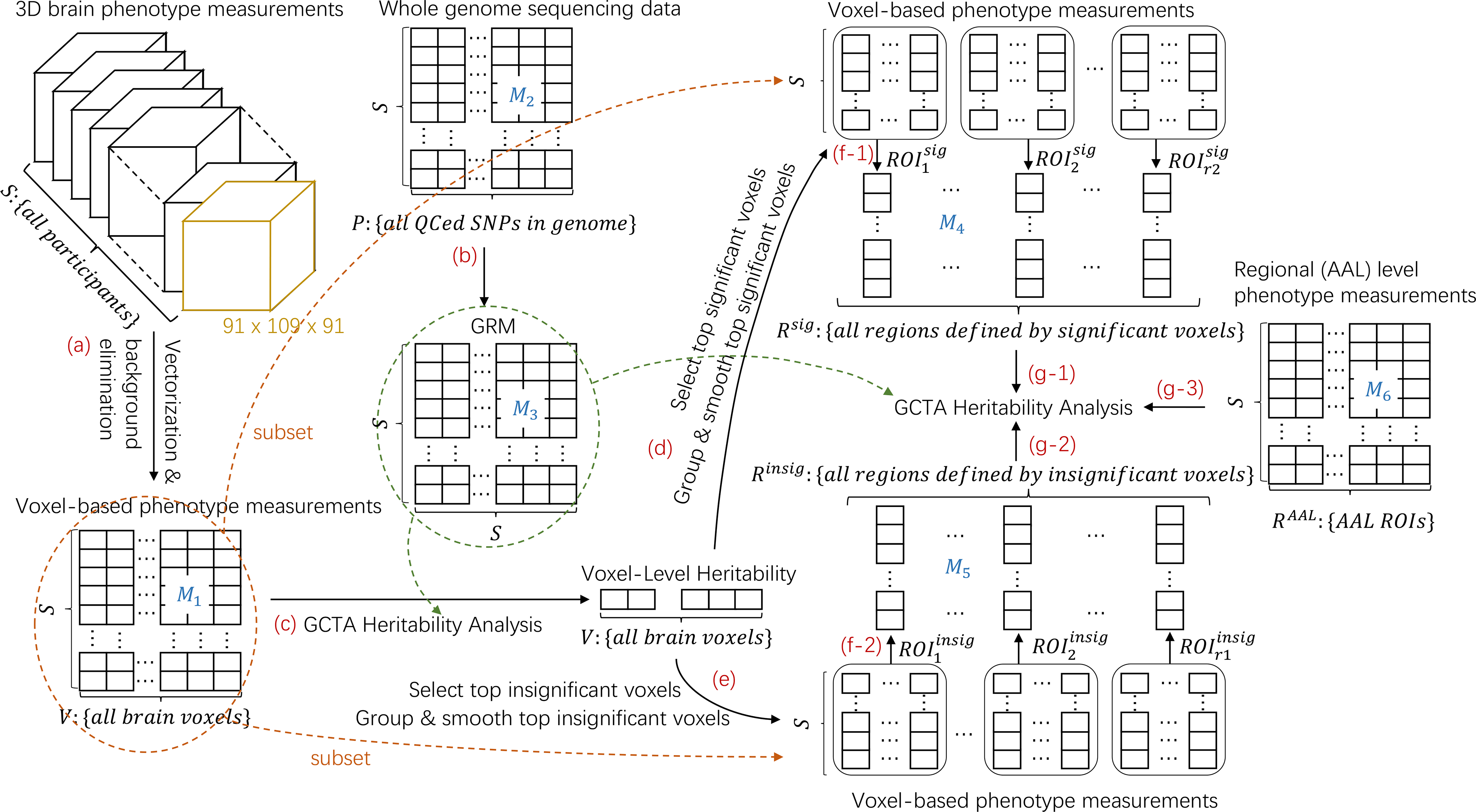
Pipeline for identifying highly heritable self-defined regions. Voxels after performing Step (d) and Step (e) form a selected subset of all brain voxels *V* meeting the corresponding selection criterion, where the number of voxels after Step (d) and Step (e) varies depending on the selection strategy (top 10%, top 20%, top 30%, or top 100%). GRM (*M*_3_) constructed by the WGS data (*M*_2_) is applied four times in defining the regions (Step (c), once) and comparing the defined regions (Steps (g-1), (g-2), and (g-3), three times) respectively.

The overall pipeline for our proposed method identifying highly heritable self-defined regions is shown in Figure 1. Starting from the three dimensional brain phenotype measurements, we first vectorize the 3D measurements into a single vector for each subject in Step (a). After filtering the background voxels defined by voxels with phenotype measurements being all 0 across all the subjects in Step (a), we are able to formulate a two dimensional voxel-based phenotype measurements matrix (*M*_1_ in Figure 1). The vectorization step is done with the voxels’ 3D coordinates preserved in a different file where the file stores the index and the spatial location for each brain voxel. In Step (b), we calculate the genetic relationship matrix (GRM) (*M*_3_ in Figure 1) using the WGS data from ADNI (*M*_2_ in Figure 1) to quantify the genetic similarity between subjects.

GCTA heritability analysis for each voxel-based QT is performed in Step (c) using the GRM and adjusted by age, sex and population structure represented by first 10 principal components. After performing the Step (c), for each voxel-based QT, we are able to calculate its heritability and the p-value. After estimation of the heritability with p-value for each voxel, we map the heritability and p-value for each voxel to the 3D brain and construct a heritability brain map and a p-value brain map. In Step (d), we filter out all the insignificant voxels and then group the top 10%, 20%, 30% and all the 100% significant voxels to construct our highly heritable self-defined regions by averaging all the voxel-level QTs within the regions which is shown in Step (f-1). Specifically, we smooth the p-value brain map using Gaussian kernel with standard deviation 0.5. The smoothing step is realized by ndimage.gaussian_filter function in scipy Python package.^20^ Then we construct a binary brain map according to the brain voxel significant level. Every voxel passing the 0.05 significant threshold (i.e., *p* < 0.05) is marked as 1 and the rest voxels are marked as 0. The ROI is constructed using connected_components function in connected-components-3d (cc3d) Python package.^21^ The detailed description of the functions can be found online.

Finally in Step (g-1), we perform the GCTA heritability analysis again to calculate the heritability for each self-defined region (*M*_4_ in Figure 1). For evaluating our self-defined regions, we perform comparative heritability analyses for the following two sets of regions. In the first comparison, we extract ROIs using a similar strategy by grouping and averaging the top 10%, 20% and 30% *insignificant* voxels together in Step (e) and Step (f-2), and then calculating the heritability for each region (*M*_5_ in Figure 1) in Step (g-2). In the second comparison, we compute the heritability for the regional level AV45 measurements defined by the AAL atlas (*M*_6_ in Figure 1) in Step (g-3).

## 5. Results and Discussion

Table 2 summarizes our comparative heritability analysis results, indicating a remarkably high heritability for our self-defined highly heritable regions (Table 2(a)) compared to the regions defined by the AAL atlas (Table 2(b)) and the regions defined by the GCTA insignificant voxels (Table 2(c)). Almost all the regions defined by our proposed method have high GCTA estimated heritability:

- 100% of those ROIs whose variations can be explained at least 90% by the genetic variations for the regions defined by the top 10% significant voxels;
- more than 90% of variations of ROIs can be explained at least 90% by the genetic variations for the regions defined by the top 20% and top 30% significant voxels;
- for those regions defined by all the significant voxels, more than 80% of ROIs can be explained at least 90% by the genetic variations.

At least 96.6% of all the regions defined by our proposed method have GCTA heritability estimates more than 80%. Among those 116 ROIs defined by the AAL atlas, on the other hand, there are only 21(18.1%) of the regions with heritability > 80%.

To further evaluate our pipeline and demonstrate our extracted regions have higher heritability measurements, we apply the pipeline onto all the insignificant brain voxels estimated by GCTA, and evaluate the heritability estimation on the regions extracted from top 10%, top 20%, and top 30% insignificant voxels. The results are shown in Table 2(c). As we expected, all the regions extracted by insignificant voxels have relatively low GCTA estimated heritability. There are less than 10% of the ROIs extracted from top 10% and top 20% of insignificant voxels have heritability greater than 50%; and less than 30% for those regions extracted from top 30% of insignificant voxels. These results re-assure that our proposed pipeline is able to robustly select highly heritable regions.

**Fig. 2:**
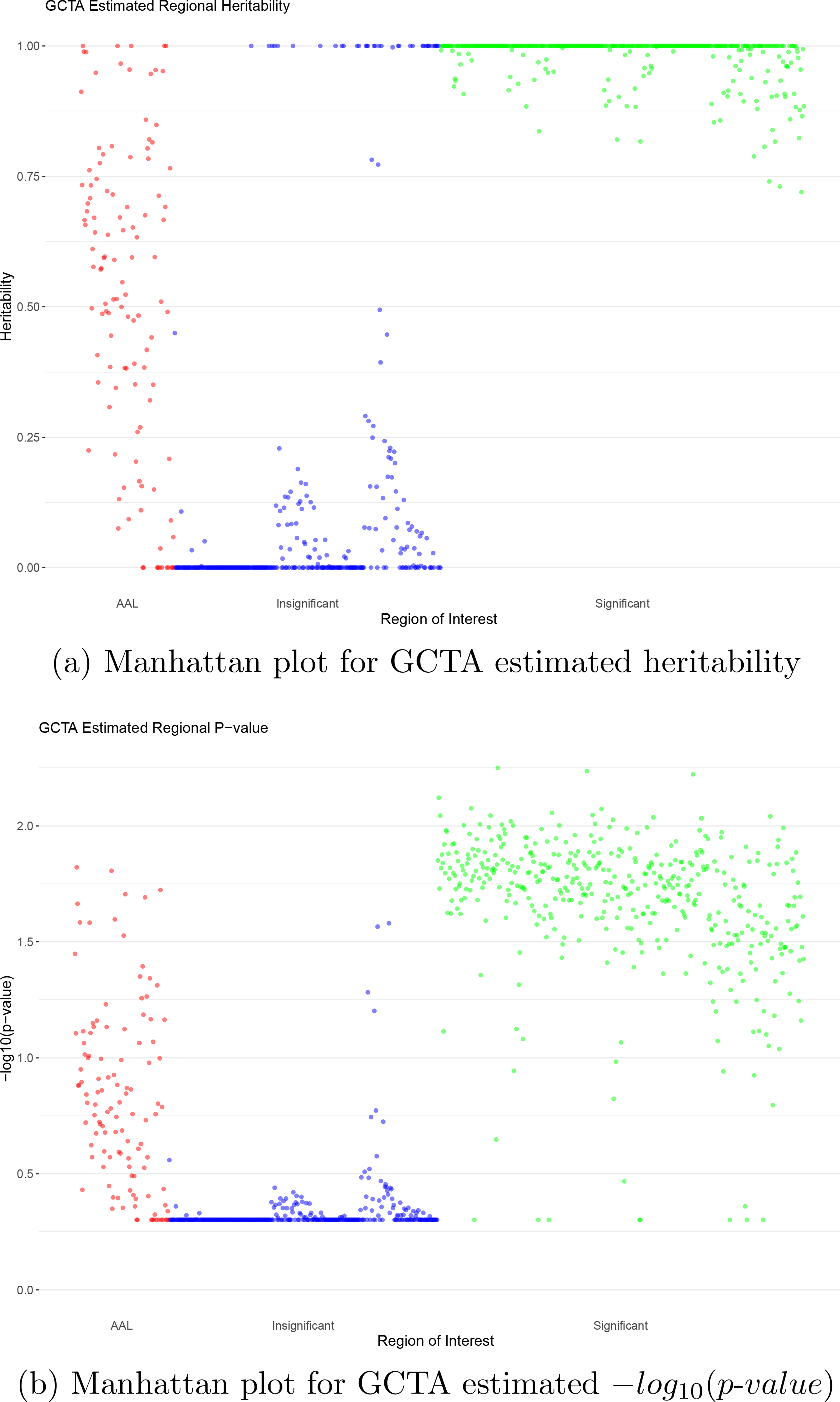
Comparison of the GCTA estimated heritability and −*log*_10_(*p-value*) for regions defined by significant voxels (green), AAL atlas (red), and insignificant voxels (blue). Regions defined by significant voxels consist of the regions defined by top 10%, top 20%, top 30%, and top 100% significant voxels. Regions defined by insignificant voxels consist of the regions defined by top 10%, top 20%, and top 30% insignificant voxels.

Figure 2 shows the distributions of GCTA heritability estimates (Figure 2a) and −*log*_10_(*p*-*value*) (Figure 2b). A clear pattern shows that the regions defined by significant voxels have an extremely high estimated heritability and −*log*_10_(*p*-*value*). Most of the regions defined by insignificant voxels have low estimated heritability and −*log*_10_(*p*-*value*). Regions defined by the AAL atlas, serving as a baseline model, have heritability estimates and −*log*_10_(*p*-*value*) widely distributed over most of the spectrum. These results align with our expectation.

**Fig. 3:**
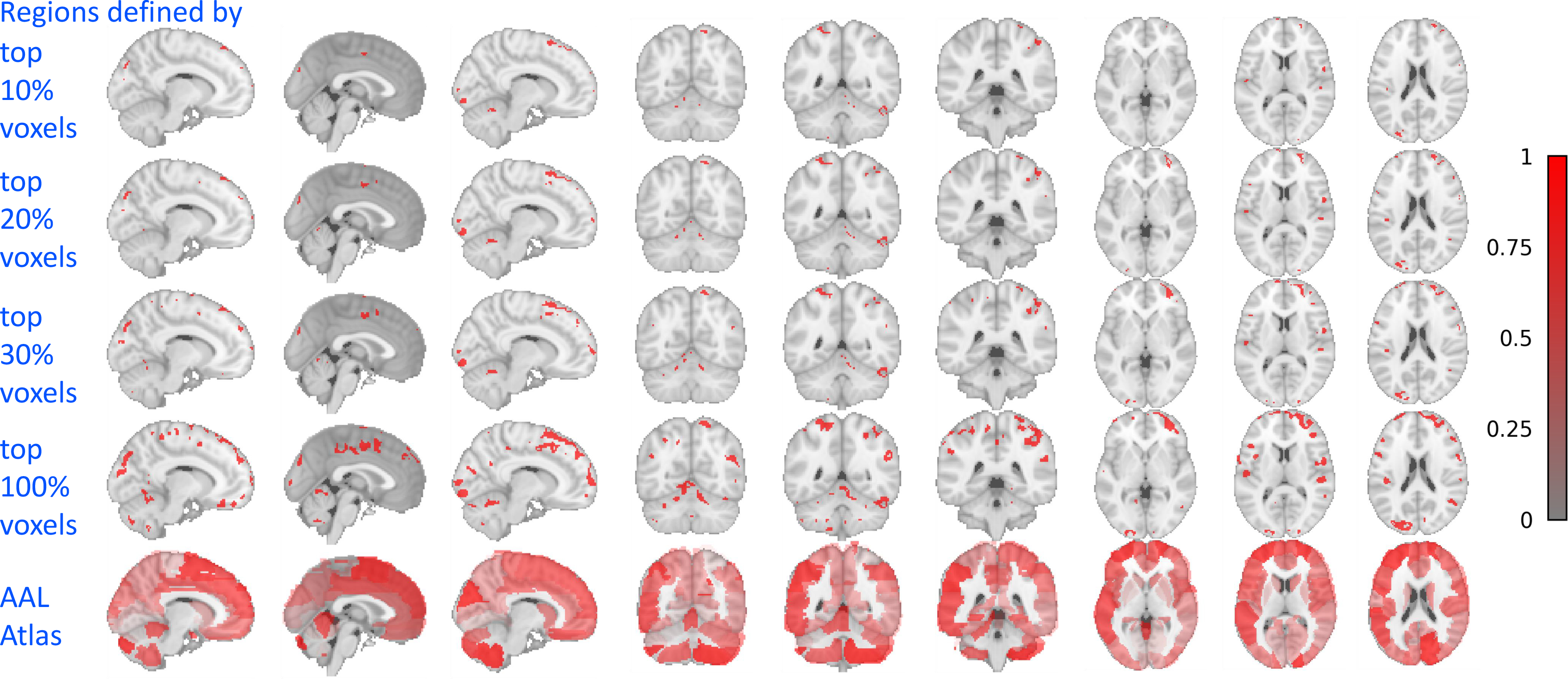
Brain heritability map for comparison between the regions defined by the top significant voxels and the regions defined by the AAL atlas. The AAL defined brain ROIs are much larger than the regions defined by our proposed pipeline, making the entire brain appear red. Although visually less striking, the proposed method has in fact yielded many more regions with high heritablity, see Table 2 and Figure 2a for details. The heritability map for regions defined by insignificant voxels are not included in this figure as their signals are few and weak, and thus the resulting brain maps are visually no difference from the background.

Figure 3 shows the heritability brain map comparing our high heritable self-defined regions and the ROIs from the AAL atlas. From top to bottom, it shows the GCTA estimated heritability for the regions extracted and smoothed using top 10%, 20%, 30% and all 100% significant voxels from voxel-level GCTA heritability estimation results. The bottom row shows the GCTA estimated heritability results for the ROIs in the AAL atlas. As shown in the figure, our self-defined regions have much higher estimated heritability than the AAL-defined regions (almost all regions extracted from our proposed method have estimated heritability close to 1). Compared to the AAL regions, our extracted regions are able to target the highly heritable parts of the brain. We also compare our highly heritable self-defined regions with the regions extracted from insignificant voxels estimated from voxel level GCTA analysis. The results show that almost all the regions extracted from insignificant voxels are not heritable (i.e., GCTA estimated heritability ≤ 10^−6^). All these observations indicate that our proposed pipeline is able to capture highly heritable regions, and thus provide valuable information to guide subsequent imaging genetic analyses.

## 6. Conclusion

In this work, we proposed a novel pipeline to define and extract highly heritable brain regions in order to guide and support the subsequent brain imaging genetic studies. We employed the widely used GCTA tool to perform SNP-based heritability estimation for imaging quantitative traits (QTs). We presented a data driven method to group highly heritable voxel-level imaging QTs according to their significance level estimated from GCTA and their spatial location. Based on voxelwise heritability estimates, we extracted brain regions containing spatially connected voxels with high heritability. We performed an empirical study on the amyloid imaging and whole genome sequencing (WGS) data from the landmark ADNI biobank. We demonstrated the regions defined by our method have much higher heritability estimates than not only the regions defined by the widely used AAL atlas but also the regions formed by voxels with low heritability. Our proposed method refines the brain imaging endophenotype constructions in light of their genetic dissection, and can yield more powerful imaging QTs to gain new insights into the phenotypic characteristics and genetic mechanisms of the brain. A potential limitation of this work is that we only performed our analysis on a discovery cohort. We are currently identifying an independent imaging genetics cohort for a future replication study to perform an unbiased evaluation of the heritable imaging traits detected here.

## Figures and Tables

**Table 1:** Participant characteristics. Total number of subjects, age, and sex are shown in this table. The mean ± sd for the age of all subjects within each diagnosis group is reported. The number of male/female subjects within each diagnosis group is also introduced.

Diagnosis	CN	MCI	AD	Overall
Number	333	384	330	1,047
Age (mean ± sd)	77.2 ± 6.8	76.4 ± 7.7	77.3 ± 7.7	76.9 ± 7.5
Sex (M/F)	149/184	226/158	189/141	564/483

**Table 2:** Statistics of heritability estimates for ROIs defined by top significant voxels, top insignificant voxels, and the AAL atlas. *ROI*_*total*_: the total number of ROIs defined by the corresponding method; *ROI*_>*a*%_: the total number(proportion among all the ROIs) of ROIs with GCTA estimated heritability > *a*% in regions defined by the corresponding method; *ROI*_<*a*%_: the total number(proportion among all the ROIs) of ROIs with GCTA estimated heritability < *a*% in regions defined by the corresponding method;

**(a) Proposed self-defined regions (extracted from top GCTA significant voxels)**						
Regions	*ROI*_*total*_	*ROI*_>90%_	*ROI*_>80%_	*ROI*_>50%_	*ROI*_<20%_	*ROI*_<10%_

Top 10% voxels	83	83(100%)	83(100%)	83(100%)	0(0%)	0(0%)
Top 20% voxels	119	117(98.3%)	119(100%)	119(100%)	0(0%)	0(0%)
Top 30% voxels	132	126(95.5%)	132(100%)	132(100%)	0(0%)	0(0%)
Top 100% voxels	118	99(83.9%)	114(96.6%)	118(100%)	0(0%)	0(0%)
**(b) AAL atlas**						
Regions	*ROI*_*total*_	*ROI*_>90%_	*ROI*_>80%_	*ROI*_>50%_	*ROI*_<20%_	*ROI*_<10%_

AAL atlas	116	14(12.1%)	21(18.1%)	64(55.2%)	22(19.0%)	16(13.8%)

**(c) Regions extracted from top GCTA insignificant voxels**						

Regions	*ROI*_*total*_	*ROI*_>90%_	*ROI*_>80%_	*ROI*_>50%_	*ROI*_<20%_	*ROI*_<10%_

Top 10% voxels	120	2(1.7%)	2(1.7%)	2(1.7%)	117(97.5%)	117(97.5%)
Top 20% voxels	116	10(8.6%)	10(8.6%)	10(8.6%)	105(90.5%)	90(77.6%)
Top 30% voxels	95	24(25.3%)	24(25.3%)	26(27.4%)	55(57.9%)	47(49.5%)
